# Associations Between Individual Health Risk Perceptions and Biomarkers of PAH Exposure Before and After PM_2.5_ Pollution in the Suburbs of Chiang Mai Province

**DOI:** 10.3390/toxics13060491

**Published:** 2025-06-11

**Authors:** Sobia Kausar, Xianfeng Cao, Sumed Yadoung, Anurak Wongta, Kai Zhou, Natthapol Kosashunhanan, Surat Hongsibsong

**Affiliations:** 1 School of Health Sciences Research, Research Institute for Health Sciences, Chiang Mai University, Chiang Mai 50200, Thailand; sobia_k@cmu.ac.th (S.K.); xianfeng_cao@cmu.ac.th (X.C.); sumed.yadoung@cmu.ac.th (S.Y.); anurak.wongta@cmu.ac.th (A.W.); 2 Environmental, Occupational Health Sciences and NCD Research Group, Research Institute for Health Sciences, Chiang Mai University, Chiang Mai 50200, Thailand; 3 School of Pharmacy and Life Sciences, Jiujiang Key Laboratory of Food Processing and Safety, Jiujiang University, Jiujiang 332005, China; zhoukaitougao@163.com

**Keywords:** PAH exposure, Chiang Mai, Thailand, BPDE, health risk perceptions, 1-OHP

## Abstract

This study examines how seasonal air pollution affects health perceptions, risk awareness, and preventive behaviors among a sample of 150 individuals, particularly within vulnerable people living in Thailand. Many participants were older adults (54.7% aged ≥ 60), female (76.7%), and had a low income (less than 10,000 THB/month (USD 295), 92.6%). Polycyclic Aromatic Hydrocarbon (PAH) exposure, as indicated by urinary 1-Hydroxypyrene (1-OHP), significantly increased during high-pollution periods (*p* < 0.001), while benzo[a]pyrene diol epoxide (BPDE) levels did not show significant changes. Farmers exhibited the highest PAH exposure (*p* = 0.018). Risk perception and preventive behavior scores rose from 0.711 to 0.748 and from 0.505 to 0.707, respectively. Notable items with high factor loadings included “burning pollutes the air and spreads pollution” (Q2.1 = 0.998) and “avoid burning of any kind” (Q4.2 = 1.007). Neurological symptoms, such as loss of consciousness, increased from 0.956 to 1.049, while respiratory problems like pneumonia went up from 0.673 to 1.07. Environmental risk knowledge-related perceptions experienced a slight decline (from 0.609 to 0.576). These results highlight the need for targeted education through community workshops and strategies like mask distribution, indoor air filtration, and early warning systems for vulnerable populations.

## 1. Introduction

Polycyclic aromatic hydrocarbons (PAHs) are a group of hazardous chemicals produced during the incomplete combustion of fuels such as petrol and diesel, and they are frequently transported by fine particulate matter (PM_2.5_) [1]. PAHs possess carcinogenic, mutagenic, and teratogenic properties, making them a significant public health concern. Exposure to PAHs can occur via inhalation of polluted air, ingestion of contaminated food, or dermal absorption [2,3].These compounds are particularly harmful to the respiratory and cardiovascular systems. Moreover, PAHs have been associated with breast cancer, although the underlying mechanisms remain unclear. Recent findings also highlight that paternal preconception exposure to PAHs may alter sperm DNA methylation, potentially affecting reproductive health [4,5,6,7].

Chiang Mai, located in Northern Thailand, experiences intense air pollution, particularly during the dry season, with PM_2.5_ and PAHs being the key pollutants [8]. During haze periods, PM_2.5_ concentrations often exceed the national annual standard of 37.5 µg/m^3^ set by the Thai Meteorological Department, with levels ranging from 21.8 to 194.0 µg/m^3^ [9,10]. A primary source of this pollution is biomass burning, which plays a significant role in the rise in both PM_2.5_ and PAH levels in the atmosphere. The composition of PAHs and their nitro derivatives (NPAHs) changes across seasons. During the dry season, biomass burning is the main contributor, whereas vehicle exhaust becomes the dominant source in the wet season [8]. Additionally, transboundary pollution (particularly from Myanmar and Laos) and secondary aerosol formation may contribute to the synchronized patterns observed during the high-PM_2.5_ season [11]. During haze episodes, the risk of inhaling excess cancer-causing PAHs in Northern Thailand is estimated at 9.3 × 10^−4^, significantly higher than during non-haze periods, where the risk is 2.5 × 10^−5^. These pollutants exceed national air quality standards and pose serious health risks, especially in areas such as Chiang Mai, where PM_2.5_ levels frequently surpass safe thresholds [9].

1-Hydroxypyrene (1-OHP) is a well-established biomarker for exposure to PAHs. Urinary levels of 1-OHP are typically higher in individuals exposed to PAHs, even in those without direct occupational or smoking exposure, making it a sensitive marker of recent exposure [12,13,14]. In addition to 1-OHP, benzo[a]pyrene (B[a]P)—a carcinogenic PAH—undergoes metabolic conversion to benzo[a]pyrene diol epoxide (BPDE), a reactive compound that binds to DNA, causing genetic damage [15,16]. BPDE levels in the blood can therefore also serve as an important biomarker for evaluating PAH exposure and its genotoxic effects [17]. Furthermore, PM_2.5_, which carries PAHs and their metabolites, is implicated in DNA damage, with the attached PAHs playing a crucial role in this process [18,19].

Despite extensive research on PM_2.5_ and PAH exposure, understanding how individuals, particularly those in suburban areas, perceive the health risks remains limited. Previous studies have primarily focused on urban versus rural populations; the perceptions, symptom experiences, and behavior of suburban communities remain underexplored. For instance, the San Pa Tong district in Chiang Mai, a suburban area reporting the highest number of respiratory symptoms, underscores the need to investigate both objective exposure levels and subjective health risk perceptions in these communities [20,21,22].

Therefore, this study aims to (1) quantify PAH exposure using biomarkers such as BPDE and 1-OHP and (2) assess residents’ perceptions of health risks related to air pollution through a validated questionnaire. This dual approach allows us to explore potential relationships between measured pollutant exposure and self-reported perceptions and symptoms in suburban Chiang Mai.

## 2. Materials and Methods

### 2.1. Study Design

**Figure 1:**
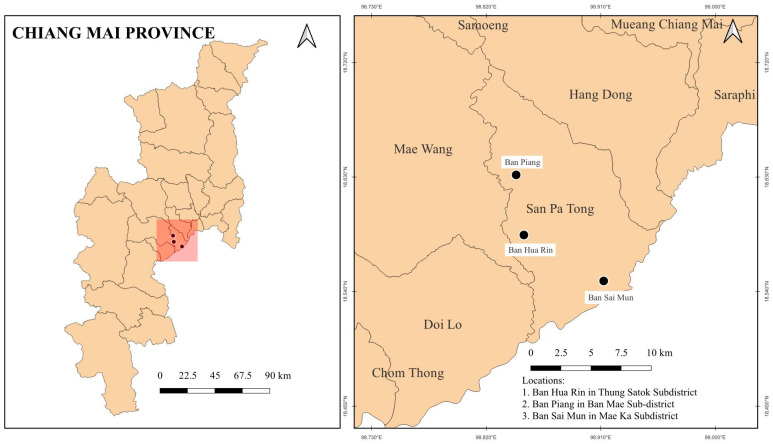
Map showing the three sampling sites located in Chiang Mai Province, Thailand.

A prospective cohort study was conducted in three locations within Chiang Mai Province: Ban Hua Rin in Thung Satok Subdistrict (18.59643° N, 98.83933° E) (Place 1), Ban Piang in Ban Mae Subdistrict (18.61202° N, 98.85485° E) (Place 2), and Ban Sai Mun in Mae Ka Subdistrict (18.56446° N, 98.94067° E) (Place 3) (Figure 1). The study consisted of two visits: the first visit occurred in December 2023, during a period of low PM_2.5_ levels (Visit 1), and the second visit took place in early April 2024, during a period of high PM_2.5_ levels (Visit 2). These locations were selected based on pre-existing air pollution data, which categorized them into areas with high and low combustion-related air pollution, ensuring a diverse range of exposure levels. A total of 150 participants were recruited, representing various occupational backgrounds. Village health volunteers facilitated participant recruitment within their respective communities. The sample size calculation was performed using G*Power software (version 3.1.9.2, Universitat Dusseldorf, Germany), with an effect size (Cohen’s d) of 0.5 (medium effect size), a power (1-β) of 0.80, and a significance level (α) of 0.05 for a two-tailed test. The effect size of 0.5 was chosen based on published literature on differences in biomarker concentrations (such as 1-OHP levels) and PM_2.5_ exposure outcomes in similar environmental health studies. This effect size allowed us to ensure that the statistical power was sufficient to detect a meaningful difference between the low- and high-PM_2.5_ seasons in our study population.

### 2.2. Ethics Approval and Consent to Participate

This research project was approved by the Human Experimentation Committee (HEC), Faculty of Associated Medical Technology, Chiang Mai University, following the Declaration of Helsinki guidelines (certificate no AMSEC-66EX-062 8/60, 3 November 2023). All procedures involving human participants adhered to ethical standards outlined in national and international guidelines. After explaining the purpose of the study, all participants provided informed consent for inclusion. The participants underwent structured interviews using a standardized questionnaire to gather detailed information. Participants were also informed of their right to withdraw from the study at any time without consequences.

### 2.3. Survey Instrument

The survey instrument followed the questionnaire by the Health Impact Assessment Division, Department of Health, Ministry of Public Health, Thailand (2021), which has been validated for reliability (Cronbach’s alpha > 0.75) into five parts, including questions on knowledge and understanding of air pollution problems, awareness of air pollution problems, experiences of being affected by air pollution problems in the past three months, preventive behaviors to reduce the impact of air pollution problems, and understanding of environmental health information. The questionnaire underwent expert review and pilot testing with 30 participants to ensure clarity and cultural relevance before its full implementation. A face-to-face questionnaire was administered to determine the perception of PM_2.5_. Demographic data were collected. Age data were gathered through interviews, while weight and height measurements were obtained via precise measurements. Socioeconomic status, smoking habits, and occupational exposure to pollutants were also documented to account for potential confounders.

### 2.4. Monitoring of PM_2.5_ Concentrations

The concentrations of PM_2.5_ were measured and recorded daily. PM_2.5_ pollution data were obtained from the Northern Thailand Air Quality Health Index (NTAQHI) website [23]. To conduct this study, we calculated the daily average.

### 2.5. Biological Sample Collection

Biological samples (urine and blood) were collected in December 2023 during the low-pollution season, and again during the early first week of April 2024, corresponding to a period of elevated PM_2.5_ concentrations in the high-pollution season. At each time point, both urine and blood samples were collected from every participant (1 sample per matrix per person per season, total of 2 urine + 2 blood samples per participant).

### 2.6. Urinary 1-Hydroxypyrene (1-OHP) Metabolite Analysis

#### 2.6.1. Urine Sample Collection

First-morning urine samples were collected from participants, stored in coded polyethylene bottles, and transported at 4 °C in zip-lock bags on ice to the Environmental and Occupational Health Sciences Research Institute, Chiang Mai University. Samples (15 mL, five tubes) were frozen at −80 °C for 1-OHP analysis. To ensure stability and reproducibility, 10% of samples were randomly selected for repeat analysis. Given that 1-OHP is a biomarker of recent exposure (typically reflecting exposures within the preceding 24–72 h) [Jongeneelen, 2001] [24], the 1-OHP levels measured in our study primarily reflect exposure during this initial high-exposure sub-period rather than representing the average exposure across the entire 90-day high season.

#### 2.6.2. Standard Chemicals and Reagents

Blank urine samples were included to ensure no background contamination in reagents. The chemicals and reagents utilized in this study included [certified reference standards for 1-hydroxypyrene (1-OHP) and 1-hydroxypyrene glucuronide (1-OHP-glu)] with a molecular weight of 218.25 g/mol and a purity of 97%, and β-glucuronidase from Helix pomatia with a concentration of 85,000 units per 10 mL, obtained from Sigma-Aldrich in Steinheim, Germany. A calibration curve was constructed using serial dilutions of standard solutions.

#### 2.6.3. Analysis of Urinary 1-Hydroxypyrene (1-OHP) Metabolites by High-Performance Liquid Chromatography with Fluorescence Detection (HPLC-FLD)

Each batch of samples was analyzed with positive and negative controls. One-OHP, known as a hydroxylated metabolite of PAHs in urine, was determined using a slight modification of the method described by Jongeneelen et al. (2001) [24]. The limits of detection (LOD) and quantification (LOQ) for 1-OHP were determined to be 0.0430 ng/mL and 0.7140 ng/mL, respectively, in accordance with EPA guidelines for analytical method validation.

#### 2.6.4. Urinary Creatinine Analysis

Urinary creatinine levels were used to adjust the dilution of the individual urine samples. Additionally, urine specific gravity was measured as an alternative correction factor to account for variations in hydration status. Jaffe’s colorimetric reaction was used to determine creatinine in the urine. Urinary 1-OHP concentrations (ng/mL) were normalized to creatinine concentrations and expressed as μmol/mol creatinine.

### 2.7. BPDE-DNA Measurement

Venous blood (3 mL) was meticulously drawn from each participant by highly trained medical laboratory technicians, adhering strictly to sterile protocols. To ensure the reliability of our results, all samples were measured in duplicate (100% intra-assay replicates) within each plate, with adjacent wells (e.g., A1-A2 and B1-B2) serving as technical replicates. The intra-assay coefficient of variation (CV) for sample replicates was consistently below 5%, demonstrating strong precision. The inter-assay CV (across plates) for the A1 position was 1.75%, further confirming assay reproducibility and reliability. Although only 10% of the samples were re-tested to confirm inter-plate consistency, the low intra- and inter-assay CVs demonstrate robust performance. The samples were then subjected to centrifugation at 4000× *g* at 4 °C for a precise duration of 15 min, effectively separating the serum. This vital serum was subsequently stored at −80 °C, preserving its integrity for further rigorous analysis.

Levels of BPDE-DNA adducts were quantified using a commercially available enzyme-linked immunosorbent assay (ELISA) kit in accordance with the manufacturer’s instructions. The ELISA kits used in this study were manufactured by Shanghai Zhuocai Biotechnology Co., Ltd. (Shanghai, China) Briefly, monoclonal antibodies specific to BPDE-DNA were pre-coated onto 96-well microplates to create a solid-phase matrix. Standards and serum samples were then added to the wells, allowing the BPDE-DNA adducts in the samples to bind to the immobilized antibodies.

Following the incubation period, horseradish peroxidase (HRP)-conjugated secondary antibodies were introduced to bind to the BPDE-DNA complexes. After a washing step to remove any unbound antibodies, a tetramethylbenzidine (TMB) substrate was added. The HRP catalyzed the reaction with the substrate, resulting in a blue color that transitioned to yellow upon the addition of an acidic stop solution. Absorbance was subsequently recorded at 450 nm using a microplate reader, from which the BPDE-DNA concentration in each sample was determined based on an eight-point standard curve. The limits of detection (LOD) and quantification (LOQ) for the BPDE-DNA ELISA assay were established at 0.1 ng/mL and 1.0 ng/mL, respectively, in accordance with the manufacturer’s specifications.

### 2.8. Statistical Analysis

Urinary 1-OHP and BPDE concentrations were analyzed using Student’s *t*-tests (StatView 4.0, Nankodo, Tokyo, Japan), with *p* < 0.05 considered statistically significant. Prior to analysis, the normality of urinary 1-OHP and BPDE data was assessed; as these data were not normally distributed, logarithmic transformation was applied before conducting parametric tests. Descriptive statistics summarized sample characteristics, pulmonary function, and PM_2.5_ perceptions, reported as numbers, percentages, means, and standard deviations (SDs). A Student’s *t*-test compared PM_2.5_ perception between middle-aged and elderly groups, while effect size calculations assessed the magnitude of differences. Exploratory factor analysis (EFA) was conducted using principal component analysis (PCA) with varimax rotation to evaluate the underlying structure of risk perception items. Factor analysis was conducted to identify latent constructs and assess the relationships between observed variables and underlying factors. The suitability of the dataset was evaluated using the Kaiser–Meyer–Olkin (KMO) measure and Bartlett’s Test of Sphericity. Factor extraction was performed using principal component analysis (PCA) or exploratory factor analysis (EFA), with the number of factors determined based on Eigenvalues and Scree plots. To enhance interpretability, factor rotation techniques such as varimax (for orthogonal rotation) and Oblimin (for oblique rotation) were applied. Factor loadings, which represent the correlation between variables and factors, were computed as standardized regression coefficients. High loadings (above 0.5) indicated strong associations, while negative loadings suggested inverse relationships. Model validation was conducted using fit indices, such as the Comparative Fit Index (CFI) and the Root Mean Square Error of Approximation (RMSEA), to ensure the robustness of the factor structure. No multiple linear regression analysis was conducted on 1-OHP or BPDE-DNA adducts. Although socioeconomic status (SES) was measured, it was not included as a confounder in statistical models. Changes in perception scores between visits were analyzed through factor analysis to assess the underlying structure of the perception data across time points. The changes in these scores were captured by evaluating the shifts in the factor loadings across visits. This method provides insight into the latent perception changes rather than directly comparing individual items. Values below the LOD (0.0430 ng/mL for 1-OHP; 0.1 ng/mL for BPDE) were noted. For sensitivity analyses, values below the LOD were imputed as LOD/√2. Main analyses were conducted both with and without these imputed values to ensure consistency.

## 3. Results

### 3.1. The Socioeconomic and Health-Related Characteristics of the Population

This table presents the socioeconomic and health-related characteristics of the sample population. The predominant age group among participants was 60 years or older (54.7%), followed by those aged 50–59 years (31.3%), with a smaller proportion aged 49 years or younger (14%). The majority were female (76.7%), with males comprising 23.3%. The primary occupations included employees and workers (53.3%), followed by farmers (27.3%) and those engaged in private business or other professions (19.3%). Regarding education, 55.3% had primary education, 37.3% had completed secondary education, and only 4.7% held a bachelor’s degree or higher. Most participants had a low monthly income, with 49.3% earning less than THB 5000 (USD 147.33), and 43.3% earning between THB 5001 (USD 147.33) and 10,000 (USD 294.66). The majority of respondents (85.3%) did not smoke, while 5.3% were current smokers and 9.3% had quit smoking. Alcohol consumption varied, with 64% abstaining and a small proportion drinking regularly (2.7%). In terms of health history, 30.9% had a family history of diabetes, 30% had relatives with cardiovascular diseases, and 17.3% had a family history of cancer. In exercise patterns, 40% exercised 3–4 times per week, while 27.3% did not engage in physical activity. Chronic illnesses were prevalent in 60.7% of participants, with the most common conditions being diabetes (49.45%), hypertension (37.6%), and hyperlipidemia (43%) (Table 1).

### 3.2. Seasonal Variation in PM_2.5_ Levels Across Subdistricts

**Figure 2:**
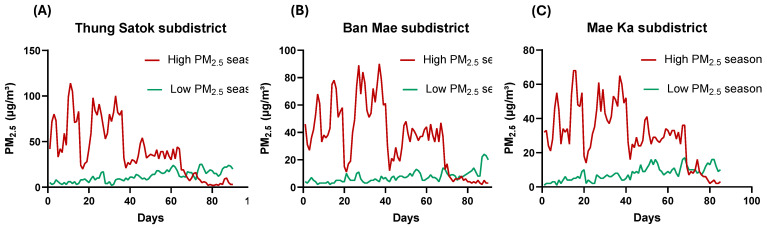
Seasonal comparison of PM_2.5_ levels across three subdistricts in Chiang Mai: (**A**) Thung Satok, (**B**) Ban Mae, and (**C**) Mae Ka. The data represent daily PM_2.5_ concentrations during the low-pollution (October–December 2023) and high-pollution (March–May 2024) seasons.

Figure 2 displays the daily PM_2.5_ levels measured at fixed air monitoring stations in three subdistricts: Thung Satok, Ban Mae, and Mae Ka. The data, obtained from the NTAQHI website, demonstrate significant seasonal variations in air quality between the high-PM_2.5_ season (March to May 2024) and the low season (October to December 2023). In Thung Satok, PM_2.5_ levels were significantly higher during the high season at 41.07 ± 28.90 µg/m^3^ compared to 11.95 ± 6.11 µg/m^3^ in the low season (*p* < 0.001, Student’s *t*-test). Similarly, in Ban Mae, the PM_2.5_ concentration increased from 6.74 ± 2.89 µg/m^3^ during the low season to 35.34 ± 24.38 µg/m^3^ in the high season (*p* < 0.001). In Mae Ka, the high season also showed a significant rise in PM_2.5_ levels, averaging 29.04 ± 17.35 µg/m^3^ compared to 7.44 ± 4.22 µg/m^3^ in the low season (*p* < 0.001). These findings confirm a substantial deterioration in air quality during the high-PM_2.5_ season across all three subdistricts.

Additional summary statistics and limit exceedance details are presented in Supplementary Table S1.

### 3.3. Comparison of 1-Hydroxypyrene (1-OHP) and BPDE Levels Across Occupations During Low (Visit 1)- and High (Visit 2)-PM_2.5_ Seasons

1-OHP and BPDE levels were analyzed based on occupation during periods of low (Visit 1) and high (Visit 2) PM_2.5_ exposure, as shown in Table 2. Throughout all occupational categories, private workers and others, employees and laborers, and farmers, 1-OHP levels rose significantly from Visit 1 to Visit 2 (*p* < 0.001), reflecting increased PAH exposure during the high-pollution period. Notably, farmers showed the most significant increase in 1-OHP, rising from 0.13 ± 0.10 to 1.06 ± 1.53 μmol/mol creatinine. Conversely, BPDE levels decreased between visits in employees and laborers (from 1.69 ± 2.42 to 0.94 ± 1.53 ng/mL, *p* = 0.02), farmers (from 1.35 ± 1.04 to 1.01 ± 1.40 ng/mL, *p* = 0.018), and the population overall (from 1.55 ± 2.06 to 0.98 ± 1.42 ng/mL, *p* = 0.0001). However, no significant change in BPDE levels was noted among private workers and others (*p* = 0.23). These results indicate occupational differences in exposure patterns and potential variations in PAH metabolism or routes of environmental exposure during the high-PM_2.5_ season. Sensitivity analyses confirmed that inclusion of imputed below-LOD values did not significantly alter mean levels, standard deviations, or primary statistical findings (*p*-values remained consistent). A breakdown of sample counts and handling strategies across LOD and LOQ thresholds, along with associated justifications and impact on statistical analyses, is presented in Supplementary Table S2.

### 3.4. Impact of Pollution Levels on Risk Knowledge and Preventive Behaviors

**Figure 3:**
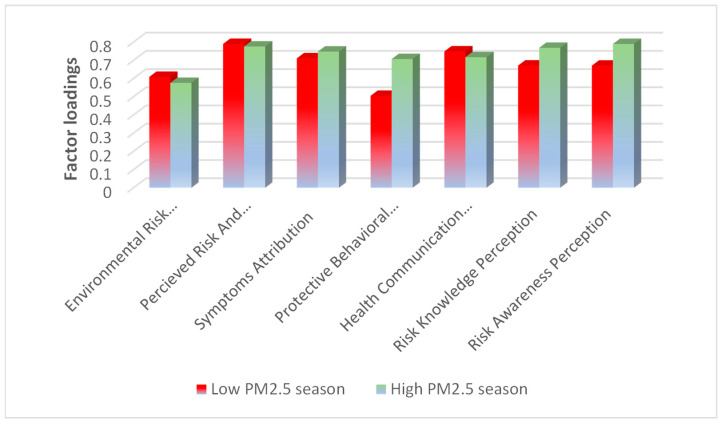
A bar chart showing changes in perception scores between the low-PM_2.5_ season and the high-PM_2.5_ season. Symptom attribution and protective behavioral intentions increased, while perceived risk and responsibility, health communication and comprehension, and environmental risk knowledge declined slightly during the period of high pollution.

During the high-pollution period (Visit 2), symptom attribution increased from 0.711 to 0.748, and protective behavioral intentions increased from 0.505 to 0.707, indicating a heightened awareness of health risks and protective behaviors. In contrast, environmental risk knowledge decreased from 0.609 to 0.576, perceived risk and responsibility decreased from 0.790 to 0.776, and health communication and comprehension decreased from 0.749 to 0.717, suggesting a decline in knowledge and awareness despite the increased exposure to pollution (Figure 3).

### 3.5. Impact of Pollution Levels on Public Awareness, Health Perception, and Preventive Behaviors

An exploratory factor analysis was performed to assess the construct validity of the questionnaire focused on perceptions concerning PM_2.5_-related risks and behaviors. This study identified multiple factors aligned with five primary dimensions: environmental risk knowledge, perceived risk and responsibility, symptom attribution, protective behavioral intentions, and health communication and comprehension, as shown in Table 3.

In the dimension of risk knowledge perception, statements including “Open burning contributes to poor air quality” (Q1.2) and “Various types of burning are responsible for the smog issue in Chiang Mai” (Q1.1) showed moderate to high factor loadings across two components. This indicates the presence of sub-factors linked to environmental and geographical awareness. Regarding perceived health effects, strong loadings were identified for claims such as “Fine particles from burning can harm the respiratory system” (Q1.6, loading = 0.864) and “Residents in polluted areas face a higher risk of lung diseases” (Q1.7, loading = 0.772).

Items related to risk awareness perception exhibited notably high loadings, especially for the statements “All types of burning make the air hotter” (Q2.2, loading = 1.063) and “Sorting garbage can solve the problem of air pollution” (Q2.5, loading = 1.084).

**Figure 4:**
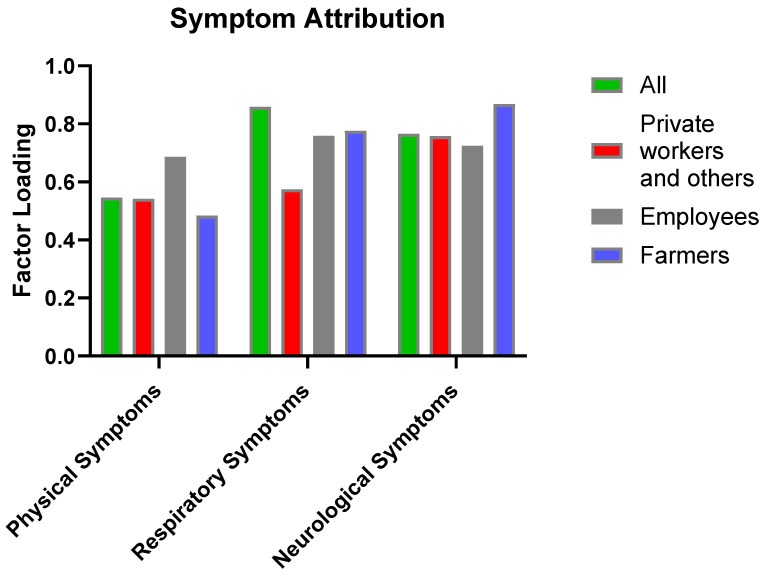
Illustrates the factor loadings for physical, respiratory, and neurological symptoms across occupational groups, highlighting that farmers and employee showed stronger attribution of symptoms, particularly neurological and respiratory, to pollution exposure.

In the directed symptom attribution dimension, neurological symptoms, including “Reported loss of consciousness” (Q3.7, loading = 0.956) and “Feeling Faint/Lightheaded” (Q3.6, loading = 0.876), exhibited the highest loadings. This was followed by respiratory symptoms such as “Coughing, sneezing, chest tightness” (Q3.3, loading = 0.851) (Figure 4).

In the context of preventive behavior perception, the phrases “Avoid any type of burning” (Q4.2, loading = 1.007) and “Turn off the engine when parked” (Q4.8, loading = 0.871) demonstrated robust links to health-conscious actions. Additionally, the environmental information perception dimension revealed significant factor loadings, particularly regarding the ability to explain and understand PM_2.5_ and its health effects (Q5.6: 0.954; Q5.3).

## 4. Discussion

Our study demonstrates that seasonal changes in PM_2.5_ pollution substantially impact biological and perceptual results among the people of Chiang Mai, especially for vulnerable groups like the elderly, those with low incomes, and agricultural workers. The combined daily PM_2.5_ concentrations at all three sample locations rose noticeably during our study area’s high-pollution season. During peak hazy conditions, the average PM_2.5_ level increased from 35 µg/m^3^ to over 120 µg/m^3^. These levels continuously surpassed Thailand’s national threshold of 37.5 µg/m^3^ [25] and the WHO’s suggested 24 h mean limit of 15 µg/m^3^ [26], underscoring the severe air pollution problems in Northern Thailand. Our study findings are consistent with previous studies, such as Amnuaylojaroen et al. [27], which reported PM_2.5_ concentrations ranging from 16 to 195 µg/m^3^ in Northern Thailand, and Othman et al. [28], which found levels between 78.3 and 209 µg/m^3^ in Chiang Mai during haze days, supporting the severity of air pollution in this region.

All subjects in our study had urine 1-OHP levels that were roughly fourfold higher during the high-PM_2.5_ season than during the low-PM_2.5_ season. This suggests that internal PAH exposure linked to seasonal air pollution has significantly increased. These findings align with those of Shen et al. [29], who found that male coke oven workers exposed to PM_2.5_ and PAHs had a 25.7-fold-higher urine 1-OHP. According to both investigations, there is a direct link between higher 1-OHP levels and increased PM_2.5_ exposure. Our results indicated that farmers showed the most significant rise in urinary 1-OHP levels compared to other occupational groups, consistent with earlier research by Ellie Darcey et al. [30] emphasizing the heightened PAH exposure risk in agriculture. Farmers often encounter greater PAH concentrations because of open-field labor, machinery use, and their closeness to biomass burning, all of which have been associated with a higher carcinogenic risk. Outdoor labor, biomass burning, and machinery use are farmers’ main sources of exposure, while indoor workers or those with less outdoor activity may be less exposed due to pollution sources. These occupation-specific differences may explain occupational disparities in urine 1-OHP and BPDE levels. Moreover, exposure frequency [16] is critical in determining risk levels, with daily outdoor activities amplifying farmers’ cumulative exposure. This is further supported by occupational studies demonstrating that workers engaged in frequent outdoor or industrial activities have significantly higher urinary 1-hydroxypyrene (1-OHP) levels, a biomarker of PAH exposure, compared to populations with lower exposure frequency. A meta-analysis reported elevated urinary 1-OHP concentrations in outdoor workers, particularly among non-smokers and individuals with specific genetic susceptibilities [31]. Similarly, steel plant workers exhibited significantly higher mean urinary 1-OHP levels (2.16 pmol/mL) than those with low or no occupational exposure (0.38 pmol/mL), underscoring the impact of occupational context on cumulative PAH exposure [32]. Additionally, nearly one-third of farmers were found to be at higher risk than trade workers when exposed to PAHs, emphasizing how occupational environments can intensify the toxic burden [33]. These findings collectively support our observation that farming as a profession may render individuals particularly vulnerable to elevated internal PAH exposure, especially during periods of high ambient PM_2.5_.

Our study found that BPDE levels showed varying responses across different occupational groups during the high-PM_2.5_ season. Private workers and others did not experience a significant change, suggesting that seasonal PM_2.5_ levels may not directly influence their PAH exposure or may reflect differences in exposure sources and timing. In contrast, employees and laborers exhibited a significant decrease in BPDE levels (*p* = 0.02), which could indicate reduced PAH exposure or alterations in metabolic processing, enhanced metabolic detoxification, or timing-related differences in biomarker detection. Interestingly, farmers did not show a significant increase in BPDE levels, despite a rise in urinary 1-OHP, suggesting that their internal PAH metabolism or exposure patterns may differ. Notably, BPDE-DNA adducts have a short half-life in circulation and are subject to inter-individual variation in detoxification and DNA repair capacity and temporal mismatch between PAH exposure and stable adduct formation [34], which may result in underestimation of BPDE levels during peak exposure. Moreover, inter-individual differences in metabolic detoxification capacity and DNA repair mechanisms may further contribute to the observed discrepancies. This highlights the complexity of PAH biomarker responses to environmental exposures across occupational settings. This lack of change may also result from inter-individual variations and possible discrepancies between blood and target tissue levels [35]. Such differences could stem from individual detoxification variability or variations in short-term exposure patterns and PAHs’ metabolic processing, as Alexandrov et al., suggested [36]. Nonetheless, numerous studies have highlighted the usefulness of this biomarker for evaluating PAH exposure across different occupational and environmental contexts.

Furthermore, our results indicate that heightened pollution levels significantly influenced risk perception, symptom attribution, and preventive behavioral intentions. During the high-pollution period, participants’ awareness of PM_2.5_-related symptoms increased (from 0.711 to 0.748), along with an increase in protective behavioral responses (from 0.505 to 0.707). This suggests that individuals became more conscious of health risks and took more protective measures, a pattern consistent with prior studies on air pollution risk perception [37]. This study proposed that perceived health risk plays a crucial role in shaping public responses to environmental hazards. When individuals attribute symptoms to pollution and perceive greater health threats, they tend to modify their behavior to reduce exposure. Studies in highly polluted regions, such as Beijing and Delhi, have shown that residents take proactive measures such as using masks, reducing outdoor activities, and advocating for policy changes when pollution levels are high [38,39]. The severity of pollution influences behavioral responses. For instance, Maung et al. [40] noted that individuals increased their use of air purifiers and adjusted their travel patterns during peak pollution seasons. However, despite the attribution of symptoms and behavioral changes, knowledge-related dimensions, such as perceived environmental risk knowledge (from 0.609 to 0.576), awareness of environmental risks (from 0.790 to 0.776), and environmental information awareness (from 0.749 to 0.717), showed a slight decline in our study. This decline could signify information fatigue, dependence on immediate protective measures, or inadequate environmental awareness, suggesting that extensive exposure to pollution does not necessarily improve long-term understanding of its sources and associated risks.

Shen et al. [29] also highlighted that elevated 1-OHP levels were associated with decreased FEV1/FVC ratios, indicating impaired lung function. In our study, while we did not directly assess lung function, participants’ perception of PM_2.5_-related health threats, particularly respiratory issues, was reflected in their subjective reports and symptom attribution. Many participants recognized air pollution as a contributor to breathing difficulties and other health symptoms, suggesting that public perception may be informed not only by visible pollution levels but also by personal or community health experiences during high-exposure periods. This alignment between scientific evidence of physiological harm and community-level risk perception underscores the importance of public education campaigns that translate complex health risks into understandable, relatable concerns.

This study boasts significant strengths that enhance its findings. A standout feature is the diverse array of occupational categories represented among the participants, including laborers and farmers. This diversity provides a robust understanding of the effects of PM_2.5_ exposure across various industries, ensuring the findings are widely applicable. By encompassing such a broad sample, this study effectively captures the nuances of PM_2.5_ impacts on different populations, which is crucial for developing targeted interventions and policies. A detailed examination of seasonal fluctuations and their impact on health outcomes is made possible by the longitudinal design, which compares seasons with low and high exposure. The findings were validated by evaluating internal PAH exposure using reliable biomarkers such as urinary 1-OHP and BPDE levels. Robust conclusions are ensured by using statistical rigor, specifically paired *t*-tests, showing elevated health risks in work settings.

It is essential to recognize the limitations of this study. First, although BPDE and 1-OHP biomarkers offer insights into PAH exposure, they fail to encompass the entire range of air pollutants. Previous studies such as Orakij et al., 2017 have shown that households using biomass fuels for cooking and heating have significantly higher indoor PAH concentrations [41]. In our future studies, we plan to consider these factors more thoroughly by including detailed data on indoor air pollution sources and lifestyle behaviors such as dietary habits, physical activity, seasonal respiratory infections, and smoking status. Additionally, integrating biomarkers like 8-iso-PGF2α [42] to assess oxidative stress could provide a more comprehensive understanding of the factors affecting BPDE levels. Second, the sample population’s generally low education level may have impacted the results regarding knowledge perception. Future studies should consider educational interventions suited to local literacy levels. Third, the questionnaire relied on single-choice responses, which restricted the depth of data. Future research should utilize Likert-scale or open-ended questions for deeper insights into public perceptions and behaviors. Moreover, while the questionnaire was pilot-tested, content and face validity were not comprehensively established, and items such as diagnosed diseases may have reduced clarity in symptom attribution. Certain symptoms reported in the questionnaire, such as pneumonia, fainting, and losing consciousness, may not have clear face validity as direct effects of ambient air pollution exposure, reflecting the complexity of self-reported health data in environmental studies. Fourth, while the questionnaire has been validated and pilot-tested, we did not comprehensively assess participants’ baseline health conditions, such as chronic respiratory or cardiovascular diseases, which may have influenced symptom reporting. Future studies should also aim to improve construct, criterion, and discriminant validity by correlating self-reported symptoms with objective clinical or biomarker data and validated external scales. Fifth, while exploratory factor analysis was applied to evaluate perception domains, the relatively small sample size may affect the generalizability of the factor structure; this constraint has been acknowledged. Sixth, the method of quantifying perception responses into a 0–1 scale from categorical data (e.g., yes/no or multiple choice) may reduce interpretability and nuance, particularly in assessing gradations of perception. Seventh, although we applied paired non-parametric tests (Wilcoxon signed-rank) to analyze perception changes between visits, subjective data remain prone to reporting bias. Eighth, since this study utilized repeated measures across two seasonal periods, an order effect and potential reporting bias may have influenced the participants’ responses. As participants become more familiar with the questionnaire over time, their responses may change. To enhance the reliability of findings, future research should explore strategies to mitigate these potential biases. Finally, while this study focuses on short-term seasonal exposure impacts, future research should consider long-term exposure effects including the risk of chronic diseases and potential genetic mutations to provide a more holistic understanding of the cumulative health impacts of air pollution.

## 5. Conclusions

In summary, our research demonstrates the notable effect of seasonal PM_2.5_ exposure on urinary 1-OHP levels, especially among occupationally exposed populations such as farmers. Although 1-OHP levels distinctly increased across all groups during the high-PM_2.5_ season, BPDE levels did not show consistent patterns, with some groups experiencing declines while others recorded rises. These results highlight the intricate relationship between PM_2.5_ exposure, PAH biomarkers, and occupational factors, indicating the necessity to explore further the health risks associated with long-term exposure and the influence of various work environments.

## Figures and Tables

**Table 1:** General characteristics of study participants (n = 150). Data are presented as frequencies and percentages. Variables include age group, gender identity, occupation, education level, monthly income, smoking and alcohol consumption behaviors, family history of chronic diseases (diabetes, cardiovascular diseases, and cancer), exercise frequency, and presence of chronic diseases.

Characteristic	n	%	Characteristic	n	%
Age group			Gender identity		
≤49 years	21	14.0	Female	115	76.7
50–59 years	47	31.3	Male	35	23.3
≥60 years	82	54.7			
Occupation			Education level		
Employees and workers	80	53.3	Primary education	83	55.3
Farmers	41	27.3	Secondary education	56	37.3
Private business/others	29	19.3	Bachelor’s or higher	11	7.3
Monthly income (THB)			Smoking status		
<5000	74	49.3	Non-smoker	128	85.3
5001–10,000	65	43.3	Current/former smoker	22	14.7
>10,000	11	7.4			
Alcohol consumption			Family history		
Never	96	64.0	Diabetes	46	30.9
Occasionally (≤4×/month)	41	27.3	Cardiovascular disease	45	30.0
Frequently (≥2×/week)	13	8.7	Cancer	26	17.3
Exercise frequency			Chronic diseases		
No exercise	41	27.3	Any chronic disease	91	60.7
1–4 times/week	83	55.3	Diabetes	46	49.5
Daily	26	17.3	High blood pressure	35	38.5
			High blood fat	40	44.0
			Asthma	9	9.9
			Other (thyroid, gout, etc.)	12	13.2

**Table 2:** Urinary 1-hydroxypyrene (1-OHP) and BPDE levels among different occupational groups at Visit 1 and Visit 2. Values are presented as mean ± S.D. *p* < 0.05 compared to Visit 1 (paired-sample *t*-test).

Occupation	1-OHP (μmol/mol Cre)			BPDE (ng/mL)		
	Low-PM_2.5_ Season Mean ± S.D.	High-PM_2.5_ Season Mean ± S.D.	*p*-Value	Low-PM_2.5_ Season Mean ± S.D.	High-PM_2.5_ Season Mean ± S.D.	*p*-Value
Private workers and others (*n* = 32)	0.15 ± 0.27	0.59 ± 0.79	<0.001	1.4 ± 2.16	1.02 ± 1.18	0.23
Employees and laborers (*n* = 77)	0.30 ± 0.71	0.90 ± 1.30	<0.001	1.69 ± 2.42	0.94 ± 1.53	0.02
Farmers (*n* = 41)	0.13 ± 0.10	1.06 ± 1.53	<0.001	1.35 ± 1.04	1.01 ± 1.40	0.018
All participants (*n* = 150)	0.22 ± 0.52	0.89 ± 1.27	<0.001	1.55 ± 2.06	0.98 ± 1.42	0.0001

**Table 3:** Exploratory factor loadings of questionnaire items related to PM_2.5_ perceptions and responses. Factor analysis was performed using principal component analysis (PCA) with varimax rotation. Only loadings ≥ 0.30 are shown. Factor Loading 1 corresponds to data from the low-PM_2.5_-exposure season (Visit 1), and Factor Loading 2 corresponds to data from the high-PM_2.5_-exposure season (Visit 2).

Dimensions		Questions	Factor Loading 1	Factor Loading 2
Environmental Risk Knowledge	The general perception of burning as a cause of pollution	Q1.1 The cause of the smog problem in Chiang Mai is all kinds of burning.	0.436	0.756
		Q1.2 Open burning is one of the causes of dirty air because there are contaminants in the air such as smoke, dust.	0.762	0.615
		Q1.3 Chiang Mai City is located in a lowland basin, so the air is not well-ventilated, causing dust to cover the city.	0.578	0.611
		Q1.4 During winter, the air is still and low, causing more pollution than at other times.	0.672	0.283
		Q1.5 Dust from construction or factories must be removed before being released into the atmosphere.	0.648	0.254
	Perceived health impacts	Q1.6 Fine particles from various types of burning can damage the respiratory system of people.	0.864	/
		Q1.7 People in areas with polluted air are more likely to suffer from lung diseases than people in other areas.	0.772	0.843
	Environmental and economic impacts	Q1.8 Air pollution causes soil and water quality to deteriorate.	0.334	0.224
		Q1.9 Smog causes traffic accidents.	0.557	0.23
		Q1.10 Smog causes poor visibility in flights, preventing planes from taking off or landing.	0.333	0.634
		Q1.11 Chiang Mai loses a lot of economic income from air pollution.	0.729	0.639
	Government response and regulations	Q1.12 People lose a lot of health care expenses from air pollution.	0.39	0.914
		Q1.13 Local officials have the power to order a ban on open burning. Those who fail to comply with the order may be subject to punishment.	0.842	0.91
Perceived Risk and Responsibility	Environmental awareness	Q2.1 Burning pollutes the air in the area where it is burned and spreads the pollution to other areas.	0.431	0.998
		Q2.2 All types of burning make the air in Mueang Chiang Mai hotter.	1.063	0.999
	Waste management	Q2.3 Reducing the amount of garbage in your home reduces air pollution.	0.495	0.7
		Q2.4 Repairing and reusing damaged items reduces air pollution.	0.974	0.386
		Q2.5 Sorting garbage before throwing it away can solve the problem of air pollution.	1.084	1.17
	Community and personal responsibility	Q2.6 Everyone should avoid burning.	/	1.103
		Q2.7 Everyone should help plant trees.	0.694	0.403
		Q2.8 Everyone should help monitor garbage burning in the community.	/	/
Symptom Attribution	Physical symptoms	Q3.1 Eye irritation.	0.792	0.312
		Q3.2 Skin irritation.	0.488	0.78
	Respiratory symptoms	Q3.3 Respiratory irritation such as coughing, sneezing, chest tightness.	0.851	0.833
		Q3.4 Feeling short of breath.	0.442	0.673
		Q3.5 Reported pneumonia diagnosis .	0.673	1.07
	Neurological symptoms	Q3.6 Feeling Faint/Lightheaded.	0.876	0.834
		Q3.7 Reported loss of consciousness.	0.956	1.049
		Q3.8 Feeling dizzy.	0.821	0.572
		Q3.9 Having poor visibility while driving.	0.496	0.605
Protective Behavioral Intentions	Preventive actions	Q4.1 Avoid outdoor exercise or stay in open areas.	0.31	/
		Q4.2 Avoid burning of any kind.	1.007	0.228
		Q4.3 Reduce the amount of household waste.	0.187	/
		Q4.4 Close the doors and windows of the house to prevent dust.	0.455	0.575
	Environmental improvements	Q4.5 Use water to wash around the house to reduce the amount of dust.	0.468	1.246
		Q4.6 Help plant trees.	0.599	0.33
	Safety and health impacts	Q4.7 Having an accident caused by smog on the roadside.	/	0.52
		Q4.8 If driving a vehicle, turn off the engine when parked.	0.871	0.905
		Q4.9 Have an annual health check to monitor health.	0.142	1.148
Health Communication and Comprehension	Knowledge and understanding	Q5.1 I can read knowledge, diagrams, or specific terms about air pollution, such as PM_2.5_, Air Quality Index (AQI), etc., with understanding.	0.357	0.937
		Q5.2 I can easily understand and know the explanations about PM_2.5_ dust from various media.	0.987	0.407
		Q5.3 I understand the explanations about how to reduce the health impacts of PM_2.5_ dust that are published in various places.	0.704	1.136
		Q5.4 I understand the causes and health impacts of the problem of fine dust in the air.	0.592	0.527
	Application and Personal Information	Q5.5 I know and understand enough about PM_2.5_ dust to be able to use it to protect my health and that of others.	0.873	0.341
	Communication and Advocacy	Q5.6 I can explain to others about the level of PM_2.5_ dust that affects health.	0.954	0.671
		Q5.7 I am open to advice on preventing and reducing the impacts of fine dust and can explain it to others.	0.776	1.003

## Data Availability

The data used and/or analyzed during the current study are available upon reasonable request from the corresponding author.

## Supplementary Materials

The following supporting information can be downloaded at https://www.mdpi.com/article/10.3390/toxics13060491/s1, Table S1: Mean±SD PM₂.₅ concentrations in three subdistricts during low (October–December 2023) and high (March–May 2024) pollution seasons. Exceedance status is shown based on the WHO air quality guideline (15 µg/m³) and Thailand’s national standard (37.5 µg/m³). Table S2: Handling of Biomarker Concentrations Near or Below Limits of Detection (LOD) and Quantification (LOQ).

## Abbreviations

The following abbreviations are used in this manuscript:

PM_2.5_	Particulate matter with a diameter less than 2.5
PAHs	Polycyclic aromatic hydrocarbons
BPDA	Benzo[a]pyrene diol epoxide
FEV1/FVC	Forced expiratory volume in the first second (FEV1) to forced vital capacity (FVC)
1-OHP	1-Hydroxypyrene
NTAQHI	Northern Thailand Air Quality Health Index
