# Supplementary material for: Associations Between Individual Health Risk Perceptions and Biomarkers of PAH Exposure Before and After PM2.5 Pollution in the Suburbs of Chiang Mai Province
Source: Toxics. 2025 Jun 11;13(6):491. doi: 10.3390/toxics13060491 (PMC12197788; doi:10.3390/toxics13060491)
Supplement: Supplementary file 1 [file toxics-13-00491-s001.zip › toxics-3611330-supplementary.pdf]

Subdistrict	Season	Mean $PM_{2.5}$ (μg/m³) ± SD	Exceeds WHO Limit (15 μg/m³)	Exceeds Thai Limit (37.5 μg/m³)
Thung Satok	Low	11.95 ± 6.11	No	No
	High	41.07 ± 28.90	Yes	Yes
Ban Mae	Low	6.74 ± 2.89	No	No
	High	35.34 ± 24.38	Yes	No
Mae Ka	Low	7.44 ± 4.22	No	No
	High	29.04 ± 17.35	Yes	No

Table S1. Mean±SD $PM_{2.5}$ concentrations in three subdistricts during low (October–December 2023) and high (March–May 2024) pollution seasons. Exceedance status is shown based on the WHO air quality guideline (15 μg/m³) and Thailand's national standard (37.5 μg/m³).

Analyte	Detection Limit (LOD)	Quantification Limit (LOQ)	Sample Category	Count (n)	Percentage (%)	Handling in Analysis	Justification / Impact on Results
**BPDE**	0.1 ng/mL	1.0 ng/mL	< LOD	13	9.00%	Excluded from quantitative analysis; retained as categorical data	Improved statistical power and sensitivity; sensitivity analyses confirmed no change to key conclusions. Low-level samples evenly distributed (p=0.82, Fisher's exact).
			LOD – LOQ	57	39.60%	Analyzed using non-parametric methods	Accounted for increased measurement uncertainty. Sensitivity analyses confirmed no change to key conclusions.
			> LOQ	74	51.40%	Core dataset for primary statistical inference (parametric tests)	Approximate normality confirmed after excluding <LOD samples.
**1-OHP**	0.0430 ng/mL	0.7140 ng/mL	< LOD	144	96.00%	Original instrument-	Sensitivity analyses (substituting with

				reported values retained (after sensitivity analyses)	LOD/2) did not materially affect means, SDs, or conclusions. Below-LOD samples evenly distributed (p=0.82, Fisher's exact).
	> LOD	6	4.00%	Included in analysis	Minimal proportion of samples above LOD.

Table S2. Handling of Biomarker Concentrations Near or Below Limits of Detection (LOD) and Quantification (LOQ).
